# Malaria

**Published:** 1900-02-17

**Authors:** 


[1] Brit. Med. Jour., Feb. 10.

### MALARIA.

Professor Celli has lately laid it down that the examination of the blood of a person suffering from malaria enables the physician not only to verify the diagnosis, but to determine the clinical variety of the disease and the stage of the febrile period, *i.e.*, to say whether the paroxysm is about to begin or is at an end. A case which occurred not long ago in Dr. Manson's practice at the Albert Docks Hospital, and has been reported by Dr. Rees,[1] serves well to illustrate the advantage which is gained by microscopic examination in such cases. The patient, a ship's master, aged 60, said to be suffering from malaria, was brought in in a state of partial unconsciousness, and only a very imperfect account could be obtained of the course of his illness. It was gathered, however, that two months before he had been for a period of five days at Dakar, in West Africa. It seemed that he had always enjoyed good health, and had left Dakar well, but that some days after leaving this port, while on his voyage from there to Hull, he became ill. On reaching Hull he was suffering from fever, and he came home to London, where he became worse, and was treated for malaria. While at home, however, he had no rigors, or vomiting, or hæmoglobinuria, but he had severe headache, was feverish, and on one occasion delirious. His mental condition was not satisfactory, and he hardly ever spoke. On admission examination of the blood showed an extreme degree of malarial infection, the parasites being very numerous, and of a malignant type. Multiply-infected corpuscles were the rule, many corpuscles containing four parasites, and a few five. The patient died the day after his admission to hospital. In regard to this case it is noteworthy that the patient was only five days in a malarious country. The date, however, when he was at Durban was about the end of the rainy season a time when ponds suitable for the breeding of the *Anopheles* would exist, and when consequently the season would be unhealthy. The importance of blood examination is also to be noted. In this case the patient was not sensible; no reliable history of the illness was forthcoming; he had practically no pyrexia, no rigors, and no sweating; and without a blood examination a diagnosis of malaria would hardly have been justifiable.

